# Th17/Treg-Related Intracellular Signaling in Patients with Chronic Obstructive Pulmonary Disease: Comparison between Local and Systemic Responses

**DOI:** 10.3390/cells10071569

**Published:** 2021-06-22

**Authors:** Juliana D. Lourenço, Walcy R. Teodoro, Denise F. Barbeiro, Ana Paula P. Velosa, Larissa E. F. Silva, Júlia B. Kohler, Alyne R. Moreira, Marcelo V. Aun, Isadora C. da Silva, Frederico L. A. Fernandes, Elnara M. Negri, Jefferson L. Gross, Iolanda F. L. C. Tibério, Juliana T. Ito, Fernanda D. T. Q. S. Lopes

**Affiliations:** 1 Laboratory of Experimental Therapeutics, Department of Clinical Medicine, School of Medicine, University of Sao Paulo, Av. Dr. Arnaldo 455—Room 1220, Sao Paulo 01246-903, Brazil; larissa_emidio@hotmail.com (L.E.F.S.); jubkohler@hotmail.com (J.B.K.); riane84@yahoo.com.br (A.R.M.); iocalvo@uol.com.br (I.F.L.C.T.); jutiyakito@hotmail.com (J.T.I.); fernandadtqsl@gmail.com (F.D.T.Q.S.L.); 2 Rheumatology Division of the Hospital das Clinicas FMUSP, Faculdade de Medicina, Universidade de Sao Paulo, Av. Dr. Arnaldo 455—Room 3124, Sao Paulo 01246-903, Brazil; walcy.teodoro@fm.usp.br (W.R.T.); apvelosa@gmail.com (A.P.P.V.); 3 Laboratory of Clinical Emergencies, Department of Clinical Medicine, School of Medicine, University of Sao Paulo, Av. Dr. Arnaldo 455—Room 3132, Sao Paulo 01246-903, Brazil; denisedna@hotmail.com; 4 Host & Defense Unit, Faculdade Israelita de Ciências da Saúde Albert Einstein School of Medicine, Av. Prof. Francisco Morato 4.293, Sao Paulo 05521-200, Brazil; marcelovivoloaun@gmail.com; 5 Clinical Immunology and Allergy Division, School of Medicine, University of Sao Paulo, Av. Dr. Enéas de Carvalho Aguiar 155—8th. Floor, Sao Paulo 05403-000, Brazil; 6 Pulmonary Division—Heart Institute of Hospital das Clinicas, School of Medicine of University of Sao Paulo, Av. Dr. Enéas Carvalho Aguiar 44, Sao Paulo 05403-000, Brazil; isadoracosta@usp.br (I.C.d.S.); frederico.fernandes@incor.usp.br (F.L.A.F.); 7 Laboratory of Cellular Biology, Department of Pathology, School of Medicine, University of Sao Paulo, Av. Dr. Arnaldo 455—Room 4349, Sao Paulo 01246-903, Brazil; emnegri@yahoo.com.br; 8 Department of Thoracic Surgery, A C Camargo Cancer Center, R. Prof Antonio Prudente 211, Sao Paulo 01509-010, Brazil; jefluizgross@yahoo.com.br

**Keywords:** COPD, Treg, Th17, STAT, intracellular signaling

## Abstract

Th17/Treg imbalance plays a pivotal role in COPD development and progression. We aimed to assess Th17/Treg-related intracellular signaling at different COPD stages in local and systemic responses. Lung tissue and/or peripheral blood samples were collected and divided into non-obstructed (NOS), COPD stages I and II, and COPD stages III and IV groups. Gene expression of *STAT3* and *-5*, *RORγt*, *Foxp3*, interleukin *(IL)-6*, *-17*, *-10*, and *TGF-β* was assessed by RT-qPCR. IL-6, -17, -10, and TGF-β levels were determined by ELISA. We observed increased *STAT3*, *RORγt*, *Foxp3*, *IL-6*, and *TGF-β* gene expression and IL-6 levels in the lungs of COPD I and II patients compared to those of NOS patients. Regarding the systemic response, we observed increased *STAT3*, *RORγt*, *IL-6*, and *TGF-β* gene expression in the COPD III and IV group and increased IL-6 levels in the COPD I and II group. *STAT5* was increased in COPD III and IV patients, although there was a decrease in *Foxp3* expression and IL-10 levels in the COPD I and II and COPD III and IV groups, respectively. We demonstrated that an increase in Th17 intracellular signaling in the lungs precedes this increase in the systemic response, whereas Treg intracellular signaling varies between the compartments analyzed in different COPD stages.

## 1. Introduction

Chronic obstructive pulmonary disease (COPD) is an inflammatory disease characterized by airway and/or alveolar abnormalities that lead to persistent airflow limitation and respiratory symptoms [1]. According to the World Health Organization, COPD is currently the third leading cause of death and it is predicted to remain so until the year 2030 [2]. The primary cause of COPD is exposure to tobacco smoke, and most patients have concomitant chronic diseases associated with COPD, which increase its morbidity and mortality [1].

The inflammatory response in COPD involves both innate and adaptive immune responses [3,4]. T lymphocytes differentiate into TCD8+ and TCD4+ cells. TCD8+ cells lead to apoptosis and cell necrosis through the release of granzyme-B and perforins and are increased in number in the lungs of COPD patients. TCD4+ cells can differentiate into different subtypes: T helper (Th)1, Th2, Th17, and regulatory T cell (Treg) [3,5,6].

TCD4+ cell subset differentiation is dependent on antigen (Ag) nature, the type of antigen presenting cells (APCs), and, most important of all, the cytokines present in the microenvironment. Cytokines are recognized by their cell surface receptors, which activate the Janus kinase–signal transducer and activator of transcription (JAK–STAT) pathway. In the presence of interleukin (IL)-6, IL-21, and IL-23, STAT3 is activated, and this is required for retinoic orphan receptor-gamma t (*RORγt*) expression and therefore for Th17 skewing. In contrast, STAT5 can regulate forkhead box P3 (*Foxp3*) gene expression, which promotes Treg differentiation and is required for Treg suppressive functions [7,8,9].

Th17 cells have been associated with COPD progression and the exacerbation of alveolar destruction and are characterized by the release of IL-17A, IL-17F, and IL-22 [6,10,11,12]. Conversely, Treg cells are responsible for regulating immune responses by suppressing inflammation and autoimmunity through the release of anti-inflammatory cytokines, such as IL-10 and transforming growth factor-β (TGF-β) [3,6].

Some studies have already evaluated Th17/Treg skewing in the lungs and/or blood samples of COPD patients [11,13,14,15,16,17]. However, most studies have evaluated the overall COPD stages or a specific disease stage, and only a few have evaluated samples from patients in the four different GOLD (Global Initiative for COPD) stages [11,16].

Additionally, there are discrepancies among the results comparing different lung compartments. Sales et al. (2017) demonstrated that Treg (Foxp3+) and IL-10+ cell numbers were decreased in the small airways of obstructed smokers compared with those of healthy smokers and control subjects, whereas an increase in IL-17+ cells was observed in both the obstructed and non-obstructed groups [18]. In contrast, the authors found an increase in Treg cells in lymphoid tissues, corroborating previous findings that showed increased Treg cell numbers in the follicles of moderate COPD patients compared with those of smokers and nonsmokers [19]. However, Chu et al. (2011) showed a decrease in Foxp3+ cells, gene, and protein expression in tissue samples from moderate and severe COPD patients [14].

Regarding intracellular signaling, upregulation of STAT3-associated gene expression was demonstrated in lung tissue samples of COPD patients, which could be a possible inflammatory sign of precancerous conditions [20]. Yew-Booth et al. (2015) evaluated the expression of p-STAT1-6 but demonstrated an increase in only p-STAT1 and p-STAT3 expression in patients with severe COPD [21]. These findings suggest that certain STAT proteins are activated at different stages of disease progression.

To the best of our knowledge, the present study is the first to evaluate the gene expression of STAT proteins and the related cytokine expression involved in Th17/Treg differentiation in patients with mild and moderate COPD in terms of both local and systemic inflammatory responses. Regarding the systemic inflammatory response, we were also able to compare the expression of these markers in COPD patients at different disease stages, including severe and very severe stages. Our findings demonstrate that an increase in Th17 intracellular signaling in the lungs precedes this increase in the systemic response, whereas Treg intracellular signaling varies between the compartments analyzed and COPD stages, with decreased IL-10 levels only in the plasma of severe COPD patients. These results reinforce the importance of intracellular signaling in the Th17/Treg imbalance and highlight the differences observed between lung and systemic responses in COPD progression.

## 2. Materials and Methods

### 2.1. Study Subjects and Case Information

We analyzed lung tissue and peripheral blood samples collected from 24 patients who underwent pulmonary surgical resection for primary or metastatic tumors from 2017 to 2019 and peripheral blood samples from 14 patients who underwent a routine pneumologist appointment.

COPD was diagnosed according to the Global Initiative for COPD (GOLD) [1]. Demographic data, medical history, smoking habits, medications, and pre- and post-bronchodilator (BD) lung function were obtained from an analysis of patient medical records.

The study subjects were divided into three groups according to their smoking habits and pulmonary function based on the following inclusion criteria:

Non-obstructed smokers (NOS) group: current and/or ex-smokers (smoking cessation >1 month) without pulmonary disease and normal pulmonary function (FEV1/FVC ≥ 0.70 and FEV1 ≥ 80%). Patients underwent pulmonary resection due to a primary or metastatic tumor. Tissue and blood samples were collected and analyzed.

COPD I and II group: current and/or ex-smokers (smoking cessation >1 month) with COPD (FEV1/FVC < 0.70) in GOLD stages I (FEV1 ≥ 80%) and II (50% ≤ FEV1 < 80%). Patients underwent pulmonary resection due to a primary or metastatic tumor. Tissue and blood samples were collected and analyzed.

To compare the systemic response markers, we also included a third group of patients diagnosed with COPD III and IV without neoplasia.

COPD III and IV group: current and/or ex-smokers (smoking cessation >1 month) with COPD (FEV1/FVC < 0.70) in GOLD stages III (30% ≤ FEV1 < 50%) and IV (FEV1 < 30%). Patients without neoplasia were assessed during their routine pneumologist appointment. Blood samples were collected and analyzed.

Patients with a clinical diagnosis of asthma, bronchiectasis, pulmonary infectious disease, α1-antitrypsin deficiency, interstitial lung disease or those who received radiotherapy treatment were excluded.

All participants provided informed written consent prior to participation. The study was conducted in accordance with the Declaration of Helsinki and was approved by the local research ethics committee (School of Medicine, University of Sao Paulo Ethics Committee, protocol 15101/2016 and 1.754.895) and by the research ethics committees of the participating institutions: A.C. Camargo Cancer Center—protocol 2306/16 and 1.881.93, where tissue and peripheral blood samples from the NOS and COPD I and II groups were collected, and the Hospital das Clinicas, School of Medicine of University of Sao Paulo (HCFMUSP), protocol 3.120.979, where peripheral blood samples from the COPD III and IV group were collected.

### 2.2. Tissue Sampling and Processing

We collected tissue samples from peripheral parenchyma and central airways as distant as possible from the resected tumor. In general, less tissue was available from central lung areas because of tumor proximity or surgical borders. The collected tissue samples were immediately frozen in liquid nitrogen and then transferred to a −70 °C freezer, where they remained until the time of analysis. Frozen tissue specimens were pulverized at negative temperatures and stored in a tube with TRIzol (Invitrogen Life Technologies, Carlsbad, CA, USA) for subsequent RT-qPCR analysis. For ELISAs, frozen tissue specimens were thawed, placed in a tube with 500 μL of PBS buffer, and homogenized using a Power Lyzer 24 (MO Bio Laboratories, Carlsbad, CA, USA).

### 2.3. Blood Sampling and Processing

Blood samples were collected in an EDTA tube (ethylenediamine tetraacetic acid) and kept at 4 °C prior to centrifugation. A portion of the blood was centrifuged for 10 min at 4 °C and 1500 rpm (453 RCF) to remove the plasma for ELISAs. After centrifugation, the plasma was transferred to another tube and stored at −70 °C until analysis.

For the separation of total leucocytes from the blood, we first lysed the red cells with lysis buffer (RCLB) (0.158 g ammonium carbonate MW: 96.09 and 12.2 g ammonium chloride MW: 53.5 in 2 L of distilled water), with an incubation sequence consisting of 10 min of incubation on ice and subsequent centrifugation at 3000 rpm (1811 RCF) for 10 min at 4 °C, which was repeated twice. Once the process was complete, the supernatant was discarded, and the isolated leucocytes were stored in a tube with TRIzol^®^ (Invitrogen, Carlsbad, CA, USA) at −70 °C until analysis.

### 2.4. Real-Time PCR

RNA extraction, reverse transcription, and quantitative real-time PCR (RT-qPCR) were performed with lung homogenates and isolated leucocytes as previously described [22]. Total RNA was extracted using TRIzol (Invitrogen Life Technologies, CA, USA), treated with DNAse (Invitrogen, Carlsbad, CA, USA), and detected at absorbance ratios of 260/280 nm and 260/230 nm. Reverse transcription into cDNA was performed using Super Script III First-Strand Synthesis Super Mix for qRT-PCR (Invitrogen, Carlsbad, CA, USA) according to the manufacturer’s instructions. Gene expression was evaluated via RT-qPCR using a StepOne Plus Real-Time PCR System (Applied Biosystems, Foster City, CA, USA) with SYBR Green as the fluorescent dye (Platinum SYBR Green qPCR Super Mix-UDG, Invitrogen, Carlsbad, CA, USA) and GAPDH as the housekeeping gene. The reaction conditions were as follows: 95 °C for 10 min, followed by 35 cycles of 95 °C for 15 s, 60 °C for 30 s and 72 °C for 30 s, and then followed by a melt curve stage of 95 °C for 15 s, 65 °C for 30 s, and 95 °C for 15 s. The relative expression was calculated based on the control group (Non-Obstructed Smokers) sample levels using the 2^−ΔΔCT^ method [23]. In this method, the Ct (cycle threshold) of the target gene corresponds to the number of amplification cycles required for the accumulated fluorescence signal of the reaction to exceed the threshold determined in the exponential phase of the curve, and the ΔCt corresponds to the number of the target gene Ct normalized by the GAPDH gene Ct.

Thus, an arbitrary number of copies of the genes of interest and housekeeping gene was calculated using the following formula:

ΔCt_target gene_ = Ct_target gene_ − Ct_GAPDH_ and ΔΔCt = ΔCt_targe gene_ − ΔCt_Control Group Mean_

The sequences of the genes of interest were acquired from http://www.ncbi.nlm.nih.gov/nucleotide (accessed on 16 May 2018) and are shown in Table 1. The sizes of the fragments generated by RT-qPCR were validated on a 1.5% agarose gel stained with ethidium bromide to confirm the size of the fragments and the specificity of amplification.

### 2.5. Quantification of Total Proteins and Cytokine Evaluation

The total protein concentration of the tissue homogenate was measured using the Bradford method (Protein Assay, Bio-Rad, Hercules, CA, USA), using a standard protein curve of bovine serum albumin (BSA; Sigma-Aldrich, San Luis, MO, USA), according to the manufacturer’s instructions. The colorimetric reaction was detected at 570 nm using an M2 spectrophotometer (Spectramax L, Molecular Devices, San Jose, CA, USA).

The levels of IL-6 (Biolegend, San Diego, CA, USA; DUO 430504-BL; limit of detection 7.8–500 pg/mL), IL-10 (R&D Systems, Minneapolis, MN, USA; DY217B-05 limit of detection 31–2000 pg/mL), IL-17A (Biolegend, San Diego, CA, USA; DUO 433914; limit of detection 3.9–250 pg/mL), and TGF-β (R&D Systems, Minneapolis, MN, USA; DY240-05 limit of detection 31–2000 pg/mL) in the supernatant of lung tissue homogenate and plasma samples were determined by ELISA, according to the manufacturer’s instructions. The reaction was read at 450 nm in the same M2 spectrophotometer listed above. IL-6, IL-10, IL-17A, and TGF-β concentrations were calculated using standard curves obtained with recombinant cytokines, and the results are expressed as pg/mL for plasma samples and corrected according to the total protein concentration and expressed as pg/mg for tissue homogenate samples.

### 2.6. Statistical Analyses

Statistical analysis was performed using SigmaStat^®^ Software 11.0 (Jandel Scientific, San Rafael, CA, USA). The Shapiro–Wilk test was used to determine the distribution of the data. Depending on the data distribution, Student’s *t*-test or Mann–Whitney test were used when analyzing two groups, and one-way ANOVA followed by Tukey’s post hoc test or Kruskal–Wallis test followed by Dunn’s test was used for comparisons among three groups. The data are presented as the median and interquartile range. We considered *p* < 0.05 to be statistically significant.

## 3. Results

A total of 38 patients were included in the study, as follows:

NOS group: a total of 14 patients were included, from which lung tissue samples (*n* = 14), blood plasma (*n* = 8), and white blood cells (*n* = 6) were collected.

COPD I and II group: a total of 10 patients were included, from which lung tissue samples (*n* = 9), blood plasma (*n* = 8), and white blood cells (*n* = 7) were collected.

COPD III and IV group: a total of 14 patients were included, from which blood plasma (*n* = 13) and white blood cells (*n* = 10) were collected.

The differences observed in the number of samples of blood plasma and white blood cells compared to the number of tissue samples in the NOS and COPD I and II groups, are due to the large volume of total blood necessary to isolate the two types of samples in the blood, which was not always possible to obtain from each individual included in the study.

The study subjects had similar baseline characteristics, and there was no significant difference in the pack-year values among the NOS, COPD I and II, and COPD III and IV groups. No individuals were exposed to any systemic immunomodulatory treatment. However, COPD III and IV patients were frequently treated with inhaled corticosteroids (ICS) (Table 2).

### 3.1. Th17 Response in Lung Tissue Homogenates

**Figure 1:**
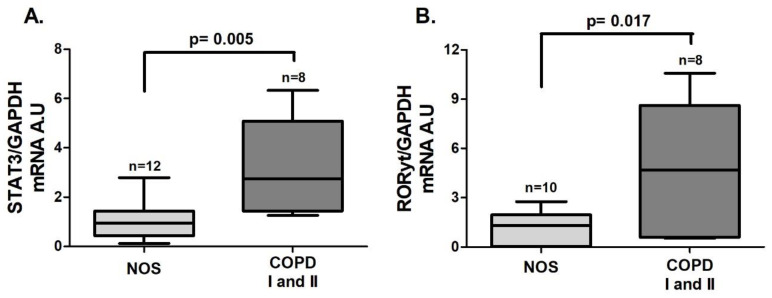
Th17 transcription factors in lung tissue samples. Th17 transcription factors gene expression in lung tissue samples: (**A**) increased *STAT3* gene expression (*p* = 0.005, *t*-test) in the COPD I and II group; (**B**) increased *RORγt* gene expression (*p* = 0.017, *t*-test) in the COPD I and II group. The data are expressed as the median and interquartile range. A.U: Arbitrary Units.

**Figure 2:**
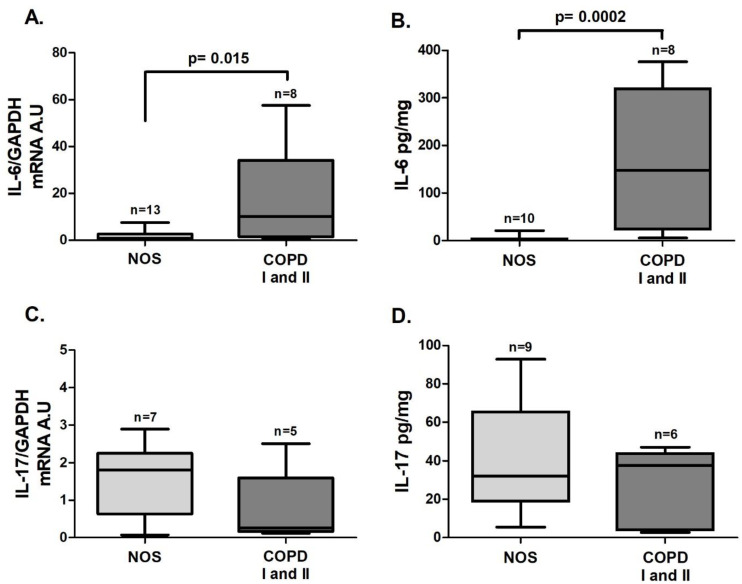
Th17-related cytokines in lung tissue samples. Th17-related cytokine expression in lung tissue samples: (**A**) increased *IL-6* gene expression (*p* = 0.015, Mann–Whitney test) and (**B**) IL-6 levels (*p* = 0.0002, Mann–Whitney test) in the COPD I and II group. No differences were observed for *IL-17* gene expression (**C**) (Mann–Whitney test) or IL-17 levels (**D**) (Mann–Whitney test) between groups. The data are expressed as the median and interquartile range. A.U: Arbitrary Units.

An increase in the gene expression of *STAT3* (Figure 1A, *p* = 0.005) and *RORγt* (Figure 1B, *p* = 0.017), both of which are transcription factors that lead to Th17 differentiation, was observed in the COPD I and II group compared to the NOS group. *IL-6* gene expression (Figure 2A, *p* = 0.015) and the IL-6 level (Figure 2B, *p* = 0.0002) were also increased in the COPD I and II group. No significant differences in *IL-17* gene expression or cytokine levels were found between the two groups (Figure 2C,D).

### 3.2. Th17 Systemic Response

**Figure 3:**
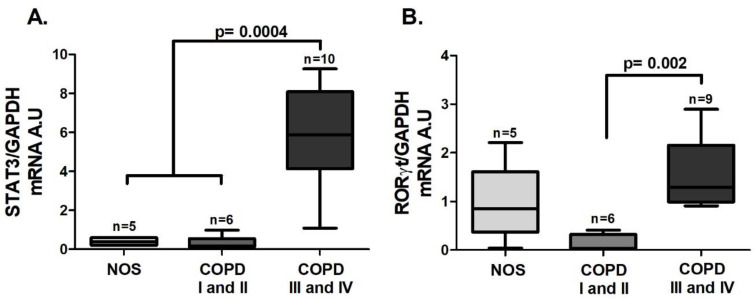
Th17 transcription factors in the systemic response. Th17 transcription factors gene expression in white blood cells: (**A**) increased *STAT3* gene expression (*p* = 0.0004, Kruskal–Wallis test) in the COPD III and IV group compared to the other groups; (**B**) increased *RORγt* gene expression (*p* = 0.002, Kruskal–Wallis test) in the COPD III and IV group compared to the COPD I and II group. The data are expressed as the median and interquartile range. A.U: Arbitrary Units.

**Figure 4:**
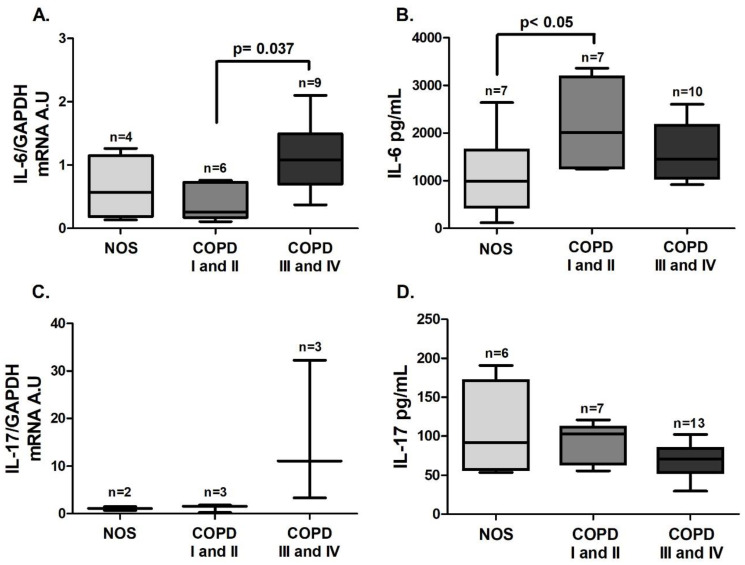
Th17-related cytokines in the systemic response. Th17-related cytokines expression in white blood cells (**A** and **C**) and plasma samples (**B** and **D**): (**A**) increased *IL-6* gene expression (*p* = 0.037, Kruskal–Wallis test) in the COPD III and IV group compared to the COPD I and II group; (**B**) increased IL-6 levels (*p* < 0.05, one-way ANOVA) in the COPD I and II group compared to the NOS group. No differences were observed for *IL-17* gene expression (**C**) (Kruskal–Wallis test) or IL-17 levels (**D**) (Kruskal–Wallis test) among the groups. The data are expressed as the median and interquartile range. A.U: Arbitrary Units.

As was observed in the tissue samples, an increase in *STAT3* (Figure 3A, *p* = 0.0004) and *RORγt* (Figure 3B, *p* = 0.002) gene expression was detected, but it was observed only in the COPD III and IV group. *IL-6* gene expression in white blood cells was increased in the COPD III and IV group compared to the COPD I and II group (Figure 4A, *p* = 0.037), and IL-6 level in blood plasma was increased in the COPD I and II group compared to the NOS group (Figure 4B, *p* < 0.05). No difference was observed in IL-17 expression in white blood cells or plasma (Figure 4C,D). Although we performed the *IL-17* gene expression analysis using the same number of samples, due to the low expression of this interleukin, only 2–3 samples of each group reached detectable values, and expression in the other samples could not be detected.

### 3.3. Treg Response in Lung Tissue Homogenates

**Figure 5:**
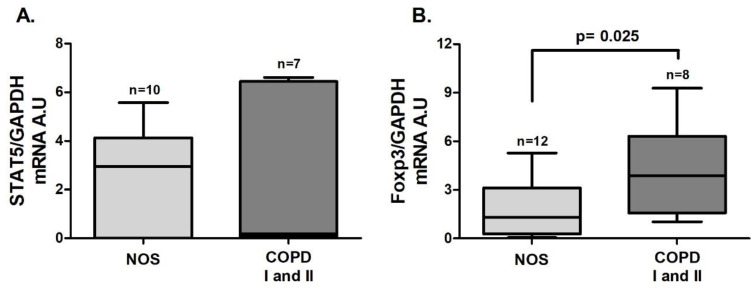
Treg transcription factors in lung tissue samples: Treg transcription factors gene expression in lung tissue samples. (**A**) No differences were observed for *STAT5* gene expression (Mann–Whitney test); (**B**) increased *Foxp3* gene expression (*p* = 0.025, *t*-test) in the COPD I and II group. The data are expressed as the median and interquartile range. A.U: Arbitrary Units.

**Figure 6:**
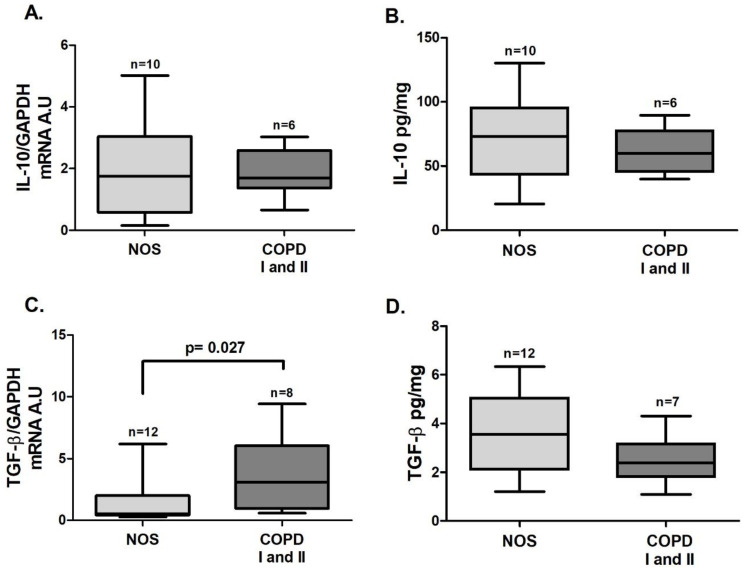
Treg-related cytokines in lung tissue samples. Treg-related cytokine expression in lung tissue samples: no differences were observed for *IL-10* gene expression (**A**, Mann–Whitney test) or IL-10 and TGF-β levels (**B**, Mann–Whitney test; **D**, *t*-test) between groups; (**C**) increased *TGF-β* gene expression (*p* = 0.027, Mann–Whitney test) in the COPD I and II group. The data are expressed as the as the median and interquartile range. A.U: Arbitrary Units.

In tissue homogenate samples, *Foxp3* (Figure 5B, *p* = 0.025) and *TGF-β* (Figure 6C, *p* = 0.027) gene expression was increased in the COPD I and II group compared to the NOS group. No differences between groups were found for *STAT5* and *IL-10* gene expression (Figure 5A and Figure 6 A) or for IL-10 and TGF-β cytokine levels (Figure 6B,D).

### 3.4. Treg Systemic Response

**Figure 7:**
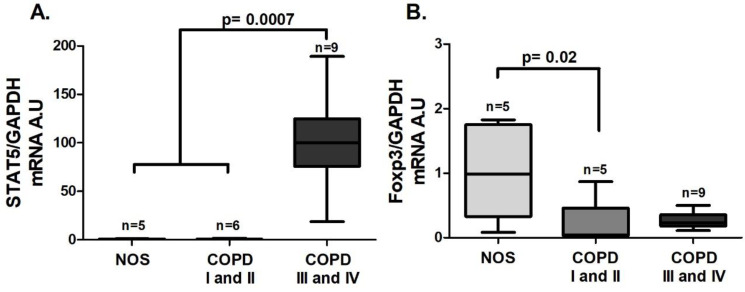
Treg transcription factors in the systemic response. Treg transcription factors gene expression in white blood cells: (**A**) increased *STAT5* gene expression (*p* = 0.0007, Kruskal–Wallis test) in the COPD III and IV group compared to the other groups; (**B**) decreased *Foxp3* gene expression (*p* = 0.02, Kruskal–Wallis test) in the COPD I and II group compared to the NOS group. The data are expressed as the median and interquartile range. A.U: Arbitrary Units.

**Figure 8:**
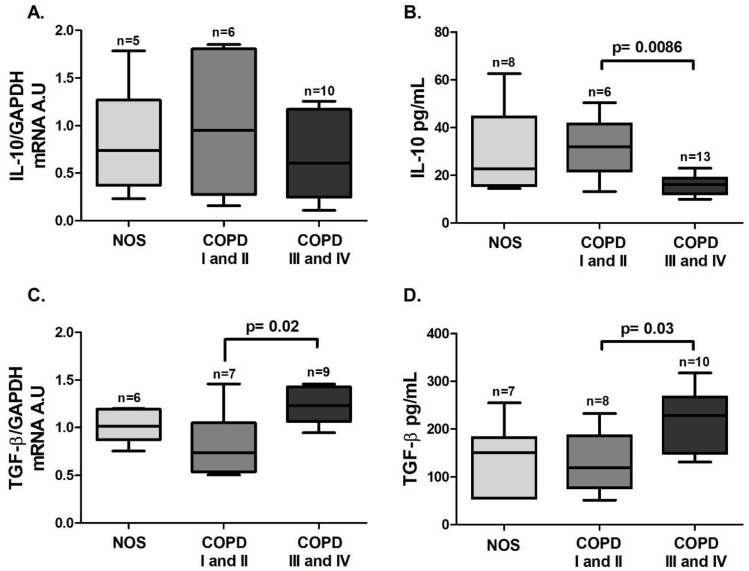
Treg-related cytokine in the systemic response. Treg-related cytokine expression in white blood cells (**A** and **C**) and plasma samples (**B** and **D**): (**A**) no differences were observed for *IL-10* gene expression (Kruskal–Wallis test) among the groups; (**B**) decreased IL-10 level (*p* = 0.0086, Kruskal–Wallis) in the COPD III and IV group compared to the COPD I and II group; (**C**) increased *TGF-β* gene expression (*p* = 0.02, Kruskal–Wallis test) and (**D**) TGF-β levels (*p* = 0.03, one-way ANOVA) in the COPD III and IV group compared to the COPD I and II group. The data are expressed as the median and interquartile range. A.U: Arbitrary Units.

An increase in *STAT5* gene expression was observed in the COPD III and IV group compared to the other groups (Figure 7A, *p* = 0.0007), and unlike the lung tissue homogenate samples, there was a decrease in *Foxp3* gene expression in the COPD I and II group compared to the NOS group (Figure 7B, *p* = 0.02). Although we could not detect a difference in *IL-10* gene expression in white blood cells among the groups (Figure 8A), there was a decrease in IL-10 level in the blood plasma of the COPD III and IV group compared to the COPD I and II group (Figure 8B, *p* = 0.0086). Additionally, an increase in both white blood cells *TGF-β* gene expression and blood plasma levels of TGF-β (Figure 8C, *p* = 0.02 and Figure 8D, *p* = 0.03) was observed in the COPD III and IV group compared to the COPD I and II group.

## 4. Discussion

Despite the large body of evidence on the role of a Th17/Treg imbalance in COPD development and progression, only a few studies have evaluated the role of different STAT signaling pathways in the skewing of these responses and the differences between lung and systemic responses.

In the present study, in both lung tissue samples and white blood cells from COPD patients, we demonstrated an increase in intracellular signaling that is related to Th17 skewing, which is mediated by transcription factors such as *STAT3* and *RORγt*. Interestingly, in the lung tissue samples, these changes could be observed in patients with mild and moderate disease (COPD I and II) when compared to smokers with no obstruction (NOS group), whereas evaluation of white blood cells revealed that these changes were present only in patients with severe and very severe stages of COPD (COPD III and IV). Yew-Booth et al. (2015) evaluated tissue samples obtained from lung transplant surgery and demonstrated an increase in phosphorylated STAT3 protein in the COPD group compared to the group of non-obstructed smokers and never-smokers [21]. In their study, COPD patients had disease stages III and IV, whereas in our study, the tissue samples were obtained from COPD patients with disease stages I and II, revealing that even in mild and moderate stages of COPD, increased *STAT3* expression is already present in lung tissue.

*IL-6* gene expression was increased in the lung samples from COPD patients who had mild and moderate disease, whereas for white blood cell samples, we detected increased expression only in patients who had severe and very severe disease. However, the IL-6 levels evaluated by ELISA were found to be increased in COPD stages I and II.

These results showed differences between local and systemic responses. Patients in earlier disease stages showed markers of Th17 skewing only in lung samples, suggesting that the systemic response occurred later. Whether systemic inflammation occurs as a result of the release of inflammatory mediators (“spill over”) from the lungs, where the inflammation would begin, or whether it is the result of some comorbidity that then affects the lungs is still uncertain [24,25,26]. Although there is evidence indicating that airway disease is associated with systemic inflammatory changes and increases in inflammation, there is still a lack of studies that confirm or disprove this hypothesis [27].

Regarding *IL-17A*, we could not verify either increased gene expression or increased levels of this interleukin in our samples. However, the increased gene expression of *RORγt* is in agreement with the increased levels of IL-6, attesting to the occurrence of Th17 skewing. Di Stefano et al. (2009) evaluated Th17-related cytokines (IL-17A, IL-22, and IL-23) in bronchial biopsies from patients with stable COPD using immunohistochemistry and/or RT-qPCR. Although IL-17A is considered the effector cytokine of Th17 cells, the authors were only able to detect an increase in IL-17-positive cells in the bronchial submucosa of COPD patients and healthy control smokers. There was no significant difference in IL-17A immunostaining of bronchial epithelium and gene expression between smokers with and those without COPD or among COPD patients at different disease stages [10]. Considering that IL-17A production is linked to structural cells such as endothelial cells [3], it likely would be easier to detect the increase in interleukin expression using techniques that evaluate specific lung compartments rather than tissue homogenates.

The evaluation of Treg skewing markers showed that, although there was an increase in *STAT5* gene expression in the white blood cells from patients in the COPD III and IV group, *Foxp3* expression was reduced in white blood cells from patients in the COPD I and II group. These changes corroborated the decrease in IL-10 levels in the COPD III and IV group. In contrast, in lung tissue samples, we observed an increase in *Foxp3* expression in the COPD I and II group, with no differences observed in *STAT5* gene expression or the level of IL-10. The lack of a difference in STAT5 protein expression was also addressed in lung tissue samples from patients with severe and very severe COPD [21]. However, the difference in Foxp3 expression among different compartments was already addressed in a study by Sales et al. (2017), which demonstrated a decrease in Foxp3+ cells in the small airways of patients with COPD compared to the control group, whereas evaluation of lymphoid tissue showed an increase in Foxp3+ cells in the non-obstructive smoker and COPD groups. Despite the increase in Foxp3+ cells in lymphoid tissues, the authors observed no difference in the density of IL-10+ cells, which could only be observed in small and large airways of COPD patients compared to non-obstructive smokers [18].

We demonstrated differences between local and systemic responses in our patient population. However, only in the plasma from patients in the severe stages of the disease did we verify decreased levels of IL-10, suggesting a failure of Treg activity.

Previous studies have demonstrated a decreased level of IL-10 in the serum of COPD stage III patients compared to patients with disease stages I and II [16] and to those with disease stages II and III, who also presented lower frequencies and numbers of *Foxp3* mRNA transcripts compared with non-obstructed smokers [17]. Sileikiene et al. (2019) evaluated the expression of Treg cells in bronchial tissue biopsies and in the blood of COPD patients [28]. The authors demonstrated decreases in the number of Treg cells in the bronchial epithelium and peripheral blood of severe/very severe COPD patients compared to those of patients with mild/moderate COPD and healthy smokers, suggesting that severe COPD is diagnosed in patients with lower levels of Tregs in the blood and respiratory tract, which corroborates our findings [28]. These authors also noted that patients with mild/moderate COPD and healthy smokers exhibited a higher number of Treg cells than the never-smoker controls, which they considered consistent with a protective role of these cells in COPD development. Additionally, increased Treg cell numbers were observed in lymphocyte follicles [19] and peripheral blood of exacerbated [13,15] and stable [15,29] COPD patients compared to healthy smokers and never-smokers. However, despite evidence of an increase in Treg cells in COPD patients, their regulatory functions may be insufficient or ineffective in regulating inflammation. According to Lane et al. (2010), the outcome of this resolution depends on pro- and anti-inflammatory influences in those patients [12], which is consistent with our findings demonstrating an imbalance towards pro-inflammatory conditions in our samples from COPD patients.

Moreover, TGF-β has been implicated as a key cytokine in the differentiation of TCD4+ cells into Th17 or Treg cells. TGF-β induces Foxp3 expression in naïve TCD4+ cells, converting them into Treg cells [30], and IL-6 together with TGF-β induces Th17 differentiation and inhibits TGF-β-induced Treg differentiation [31]. In addition to its immunomodulatory function, TGF-β has fibrogenic activity, and its increase may result in fibrosis of the airways and contribute to airflow limitation in small-airway diseases, such as COPD [32]. TGF-β1 can increase extracellular matrix (ECM) production and myofibroblast differentiation in pulmonary fibroblasts from individuals with COPD [33], while inhibition of TGF-β signaling, both in vitro and in vivo, can protect the lungs from morphological changes, lung function impairment, and injury [34,35]. In our study, increased *TGF-β* was observed in lung tissue from COPD patients with disease stages I and II and in the white blood cells and plasma of patients with disease stages III and IV. These increases may be related to the observed increases in IL-6 levels, leading to Th17 differentiation and impaired lung function in the COPD groups.

Our study has some limitations, such as the lack of lung tissue specimens from subjects in the COPD III and IV group and the fact that these patients did not present any neoplastic disease. Regarding the systemic analyses, we performed comparisons among groups with and without neoplastic disease. It would be reasonable to postulate that individuals with neoplastic disease could present greater levels of inflammatory mediators. In a review, Marshall et al. (2016) showed different studies attesting an increase not only in the Th17 response but also in the Treg response in patients presenting a lung tumor [36]. However, we observed an opposite pattern of response in this present study. The increase in *RORyt* and *STAT3*, both Th17 mediators, and in *STAT5*, a Treg-related mediator, was observed only in patients with COPD III and IV, who did not present neoplastic disease. These findings were obtained along with decreased values of *Foxp3* expression in both COPD groups. In addition, the samples obtained from patients who also had neoplasia (NOS and COPD I and II groups) had not received any systemic treatment prior to surgery.

Also, although many individuals received ICS treatment, which could interfere with pulmonary tissue findings, this treatment could not explain our systemic biomarker findings. Furthermore, most cytokines act on cells close to their cell of origin (paracrine action), and in some cases, enough of the cytokines may be produced so that a significant amount may enter the circulation and act at a distance (endocrine action) [37]. Thus, it can be hard to correlate circulating blood measurements with tissue sample measurements.

In summary, our results demonstrate an increase in Th17 intracellular signaling in lung tissue samples starting in the early stages of COPD. However, intracellular signaling associated with the Treg response seems to vary depending on the compartment analyzed, and the failure of Treg function is more evident in advanced stages of the disease.

## 5. Conclusions

Intracellular signaling associated with Th17 skewing is present starting in the early stages of COPD development, and Th17 markers can be detected in lung samples from COPD patients with disease stages I and II, whereas in terms of the systemic response, these changes were observed only in COPD patients with disease stages III and IV. Despite the increase in markers of Treg skewing, such as *Foxp3* in COPD I and II lung tissue samples and *STAT5* in white blood cells from COPD III and IV patients, decreases in IL-10 levels were detected only in the samples from patients with severe disease. These results suggest that a failure of Treg cell function plays a pivotal role in COPD progression and severity.

## Figures and Tables

**Table 1:** Forward and reverse sequence of primers used for real-time PCR.

PRIMER	FORWARD SEQUENCE 5′-3′	REVERSE SEQUENCE 3′-5′
**STAT3**	CGGACTGGATCTGGGTCTTA	CCTTTGGAACGAAGGGTACA
**STAT5**	GTGGACGATGACAACCACAG	CTGAACAACTGCTGCGTGAT
**RORγt**	TGAGAAGGACAGGGAGCCAA	CCACAGATTTTGCAAGGGATCA
**FOXP3**	CAGCACATTCCCAGAGTTCCTC	GCGTGTGAACCAGTGGTAGATC
**IL-6**	CCTGAGAAAGGAGACATGTAA	GGCAAGTCTCCTCATTGAATCC
**IL-17A**	CTTGTCCTCAGAATTTGGGCATCC	GACTCCTGGGAAGACCTCATTGG
**IL-10**	AAGCCTGACCACGCTTTCTA	ATGAAGTGGTTGGGGAATGA
**TGF-β**	GGAAATTGAGGGCTTTCGCC	AGTGAACCCGTTGATGTCCA
**GAPDH**	TGCCAAATATGATGACATCAAGAA	GGAGTGGGTGTCGCTGTTG

**Table 2:** Characteristics of the patients: gender, age, smoking habit, and pulmonary function values.

	NOS	COPD I and II	COPD III and IV
**Center (no. of patients)**			
**AC Camargo Cancer Center**	14	10	0
**HCFMUSP**	0	0	14
**Age (years)**	61.29 ± 9.47	68.44 ± 4.44	61 ± 7.78
**Male/Female**	5/9	7/3	6/8
**Smokers/Ex-smokers**	4/10	4/6	2/12
**Pack-years**	30.01 ± 22.35	37.40 ± 24.71	47.53 ± 35.39
**Inhaled Corticosteroids**	1/14	3/10	14/14 *
**FEV_1_% pred**	95.36 ± 13.33	71.10 ± 14.65 #	29.21 ± 8 *
**FEV_1_/FVC %**	77.21 ± 5.67	64.4 ± 4.69 #	45.57 ± 10.99 *
**FEV_1_% pred (Post-BD)**	97.25 ± 12.8	76 ± 15.22 #	31.36 ± 9.07 *
**FEV_1_/FVC % (Post-BD)**	77.93 ± 4.89	65.5 ± 4.32 #	44.43 ± 11.63 *

The data are presented as the mean ± standard deviation. NOS: Non-obstructed smokers; COPD I and II: subjects with a diagnosis of chronic obstructive pulmonary disease (COPD) stage I or II; COPD III and IV: subjects with a diagnosis of COPD stage III or IV; HCFMUSP: Hospital das Clinicas, School of Medicine of University of Sao Paulo; FEV_1_: forced expiratory volume in 1 s; % pred: % predicted; FVC: forced vital capacity; BD: bronchodilator. * *p* < 0.0001, COPD III and IV group compared with the other groups; # *p* < 0.001, COPD I and II group compared with the NOS group.

## Data Availability

The data presented in this study are available within the article.

## References

[1] Global Initiative for Chronic Obstructive Lung Disease (GOLD) (2020). Global Strategy for Diagnosis, Management and Prevention of COPD.

[2] WHO (2018). Projections of Mortality and Causes of Death, 2016 to 2060.

[3] Brusselle G.G., Joos G.F., Bracke K.R. (2011). New insights into the immunology of chronic obstructive pulmonary disease. Lancet.

[4] Cosio M.G., Saetta M., Agusti A. (2009). Immunologic aspects of chronic obstructive pulmonary disease. N. Engl. J. Med..

[5] Barnes P.J. (2014). Cellular and molecular mechanisms of chronic obstructive pulmonary disease. Clin. Chest Med..

[6] Caramori G., Casolari P., Barczyk A., Durham A.L., Di Stefano A., Adcock I. (2016). COPD immunopathology. Semin. Immunopathol..

[7] Williams L.M., Rudensky A.Y. (2007). Maintenance of the Foxp3-dependent developmental program in mature regulatory T cells requires continued expression of Foxp3. Nat. Immunol..

[8] Seif F., Khoshmirsafa M., Aazami H., Mohsenzadegan M., Sedighi G., Bahar M. (2017). The role of JAK-STAT signaling pathway and its regulators in the fate of T helper cells. Cell Commun. Signal..

[9] Burchill M.A., Yang J., Vogtenhuber C., Blazar B.R., Farrar M.A. (2007). IL-2 Receptor β-Dependent STAT5 Activation Is Required for the Development of Foxp3 + Regulatory T Cells. J. Immunol..

[10] Di Stefano A., Caramori G., Gnemmi I., Contoli M., Vicari C., Capelli A., Magno F., D’Anna S.E., Zanini A., Brun P. (2009). T helper type 17-related cytokine expression is increased in the bronchial mucosa of stable chronic obstructive pulmonary disease patients. Clin. Exp. Immunol..

[11] Zhang L., Cheng Z., Liu W., Wu K. (2013). Expression of interleukin (IL)-10, IL-17A and IL-22 in serum and sputum of stable chronic obstructive pulmonary disease patients. COPD J. Chronic Obstr. Pulm. Dis..

[12] Lane N., Robins R.A., Corne J., Fairclough L. (2010). Regulation in chronic obstructive pulmonary disease: The role of regulatory T-cells and Th17 cells. Clin. Sci..

[13] Jin Y., Wan Y., Chen G., Chen L., Zhang M.Q., Deng L., Zhang J.C., Xiong X.Z., Xin J.B. (2014). Treg/IL-17 ratio and Treg differentiation in patients with COPD. PLoS ONE.

[14] Chu S., Zhong X., Zhang J., Lao Q., He Z., Bai J. (2011). The expression of Foxp3 and ROR gamma t in lung tissues from normal smokers and chronic obstructive pulmonary disease patients. Int. Immunopharmacol..

[15] Li X.N., Pan X., Qiu D. (2014). Imbalances of Th17 and Treg cells and their respective cytokines in COPD patients by disease stage. Int. J. Clin. Exp. Med..

[16] Silva B.S.A., Lira F.S., Ramos D., Uzeloto J.S., Rossi F.E., Freire A.P.C.F., Silva R.N., Trevisan I.B., Gobbo L.A., Ramos E.M.C. (2018). Severity of COPD and its relationship with IL-10. Cytokine.

[17] Wang H., Ying H., Wang S., Gu X., Weng Y., Peng W., Xia D., Yu W. (2015). Imbalance of peripheral blood Th17 and Treg responses in patients with chronic obstructive pulmonary disease. Clin. Respir. J..

[18] Sales D.S., Ito J.T., Zanchetta I.A., Annoni R., Aun M.V., Ferraz L.F.S., Cervilha D.A.B., Negri E., Mauad T., Martins M.A. (2017). Regulatory T-Cell Distribution within Lung Compartments in COPD. COPD J. Chronic Obstr. Pulm. Dis..

[19] Plumb J., Smyth L.J.C., Adams H.R., Vestbo J., Bentley A., Singh S.D. (2009). Increased T-regulatory cells within lymphocyte follicles in moderate COPD. Eur. Respir. J..

[20] Qu P., Roberts J., Li Y., Albrecht M., Cummings O.W., Eble J.N., Du H., Yan C. (2009). Stat3 downstream genes serve as biomarkers in human lung carcinomas and chronic obstructive pulmonary disease. Lung Cancer.

[21] Yew-Booth L., Birrell M.A., Lau M.S., Baker K., Jones V., Kilty I., Belvisi M.G. (2015). JAK-STAT pathway activation in COPD. Eur. Respir. J..

[22] Junqueira J.J.M., Lourenço J.D., da Silva K.R., de Brito Cervilha D.A., da Silveira L.K.R., Correia A.T., de França Silva L.E., Teodoro W.R., Tibério I.d.F.L.C., Barbosa A.P. (2020). Decreased Bone Type I Collagen in the Early Stages of Chronic Obstructive Pulmonary Disease (COPD). COPD J. Chronic Obstr. Pulm. Dis..

[23] Livak K.J., Schmittgen T.D. (2001). Analysis of relative gene expression data using real-time quantitative PCR and the 2-ΔΔCT method. Methods.

[24] Agustí A., Edwards L.D., Rennard S.I., MacNee W., Tal-Singer R., Miller B.E., Vestbo J., Lomas D.A., Calverley P.M.A., Wouters E. (2012). Persistent systemic inflammation is associated with poor clinical outcomes in copd: A novel phenotype. PLoS ONE.

[25] Agustí A. (2007). Systemic effects of chronic obstructive pulmonary disease: What we know and what we don’t know (but should). Proc. Am. Thorac. Soc..

[26] Barnes P.J., Celli B.R. (2009). Systemic manifestations and comorbidities of COPD. Eur. Respir. J..

[27] Sinden N.J., Stockley R.A. (2010). Systemic inflammation and comorbidity in COPD: A result of “overspill” of inflammatory mediators from the lungs? Review of the evidence. Thorax.

[28] Sileikiene V., Laurinaviciene A., Lesciute-Krilaviciene D., Jurgauskiene L., Malickaite R., Laurinavicius A. (2019). Levels of CD4+ CD25+ T regulatory cells in bronchial mucosa and peripheral blood of chronic obstructive pulmonary disease indicate involvement of autoimmunity mechanisms. Adv. Respir. Med..

[29] Li H., Liu Q., Jiang Y., Zhang Y., Zhang Y., Xiao W. (2015). Disruption of Th17/Treg balance in the sputum of patients with chronic obstructive pulmonary disease. Am. J. Med. Sci..

[30] Chen W., Konkel J.E. (2010). TGF-β and ‘adaptive’ Foxp3+ regulatory T cells. J. Mol. Cell Biol..

[31] Kimura A., Kishimoto T. (2010). IL-6: Regulator of Treg/Th17 balance. Eur. J. Immunol..

[32] Yang Y.C., Zhang N., Van Crombruggen K., Hu G.H., Hong S.L., Bachert C. (2012). Transforming growth factor-beta1 in inflammatory airway disease: A key for understanding inflammation and remodeling. Allergy Eur. J. Allergy Clin. Immunol..

[33] Baarsma H.A., Spanjer A.I.R., Haitsma G., Engelbertink L.H.J.M., Meurs H., Jonker M.R., Timens W., Postma D.S., Kerstjens H.A.M., Gosens R. (2011). Activation of WNT/ β-csatenin signaling in pulmonary fibroblasts by TGF-β 1 is increased in chronic obstructive pulmonary disease. PLoS ONE.

[34] Podowski M., Calvi C., Metzger S., Misono K., Poonyagariyagorn H., Lopez-Mercado A., Ku T., Lauer T., McGrath-Morrow S., Berger A. (2012). Angiotensin receptor blockade attenuates cigarette smoke—Induced lung injury and rescues lung architecture in mice. J. Clin. Investig..

[35] Wang Z., Fang K., Wang G., Guan X., Pang Z., Guo Y., Yuan Y., Ran N., Liu Y., Wang F. (2019). Protective effect of amygdalin on epithelial–mesenchymal transformation in experimental chronic obstructive pulmonary disease mice. Phyther. Res..

[36] Marshall E.A., Ng K.W., Kung S.H.Y., Conway E.M., Martinez V.D., Halvorsen E.C., Rowbotham D.A., Vucic E.A., Plumb A.W., Becker-Santos D.D. (2016). Emerging roles of T helper 17 and regulatory T cells in lung cancer progression and metastasis. Mol. Cancer.

[37] Abbas A.K., Lichtman A., Pillai S. (2018). Cellular and Molecular Immunology.

